# Summary data of potency and parameter information from semi-mechanistic PKPD modeling of prolactin release following administration of the dopamine D_2_ receptor antagonists risperidone, paliperidone and remoxipride in rats

**DOI:** 10.1016/j.dib.2016.07.060

**Published:** 2016-08-06

**Authors:** Amit Taneja, An Vermeulen, Dymphy R.H. Huntjens, Meindert Danhof, Elizabeth C.M. De Lange, Johannes H. Proost

**Affiliations:** a Division of Pharmacokinetics, Toxicology and Targeting, Groningen Research Institute of Pharmacy, University of Groningen, Antonius Deusinglaan 1, 9713 AV Groningen, The Netherlands; b Clinical Pharmacology and Pharmacometrics, Janssen Research & Development, A Division of Janssen Pharmaceutica NV, Beerse, Belgium; c Department of Pharmacology, Leiden Academic Center for Drug Research, Leiden University, The Netherlands

## Abstract

We provide the reader with relevant data related to our recently published paper, comparing two mathematical models to describe prolactin turnover in rats following one or two doses of the dopamine D_2_ receptor antagonists risperidone, paliperidone and remoxipride, “A comparison of two semi-mechanistic models for prolactin release and prediction of receptor occupancy following administration of dopamine D_2_ receptor antagonists in rats” (Taneja et al., 2016) [1]. All information is tabulated. Summary level data on the in vitro potencies and the physicochemical properties is presented in Table 1. Model parameters required to explore the precursor pool model are presented in Table 2. In Table 3, estimated parameter comparisons for both models are presented, when separate potencies are estimated for risperidone and paliperidone, as compared to a common potency for both drugs. In Table 4, parameter estimates are compared when the drug effect is parameterized in terms of drug concentration or receptor occupancy.

**Specifications Table**

**Table:** 

Subject area	Pharmacology
More specific subject area	Neuropsychopharmacology
Type of data	Tables
How data was acquired	Experimental study in male wistar rats, as described below
Data format	Processed tabulated data
Experimental factors	Plasma samples were collected for bioanalysis of risperidone, paliperidone, and remoxipride using an on-line solid phase extraction with liquid chromatography – tandem mass spectrometry method. Serum prolactin levels were measured using an enzyme linked immunosorbent assay technique.
Experimental features	All animal procedures were performed at Leiden University, in accordance with Dutch laws governing animal experimentation. Male Wistar rats, received single intravenous doses of risperidone (2 mg/kg, *n*=16) or paliperidone (0.5 mg/kg, *n*=21). Plasma drug concentrations as well as plasma prolactin levels were measured at pre-dose and at serial intervals post-dose. In another study, remoxipride was administered to rats either as a single intravenous dose of 4, 8 or 16 mg/kg (*n*=10) remoxipride or two doses of 3.8 mg/kg at varying dosing intervals. Blood samples were serially collected. Plasma concentrations of the drugs as well as prolactin were assayed using validated analytical methods.
Data source location	Department of Pharmacology, Leiden Academic center for Drug Research, Leiden.
Data accessibility	The data is within this article.

**Value of the data**

Data can be used

• To compare experimental findings in literature with our model-based approach.
• As prior information, especially when the available data is scarce.
• For exploratory modeling.
• For translation from rat to humans.

## 1 Data

The information is presented in 4 tables. Table 1 presents the in vitro inhibition constant (*KI*) values in rat and humans and physicochemical characteristics of the antipsychotics risperidone, paliperidone and remoxipride. Table 2 presents the pharmacokinetic–pharmacodynamic model parameters used to perform exploratory model simulations of the precursor pool model, as referred to in Section 3.2, Fig. 5 of Taneja et al. [1]. Table 3 presents the model parameters assuming equal or different potency of risperidone and paliperidone. Table 4 presents the model parameters obtained with different parameterizations, assuming either unbound drug concentration or dopamine D_2_ receptor occupancy as the driving force for drug effect.

## 2 Experimental design, materials and methods

Details of the experimental procedures have been described previously [1], [5], [6].

## Figures and Tables

**Table 1:** Overview of literature *KI* values and physicochemical characteristics of risperidone, paliperidone and remoxipride.

	**Risperidone**	**Paliperidone**	**Remoxipride**
***KI*****values** (nM)			
Rat⁎	2.55	2.74	370.66
Human⁎	2.18	2.08	165.75
Human⁎⁎	4.9 / 6	NA	243 / 125
**Physicochemical characteristics**			
Protein binding % (rat)⁎	88.2	74.7	20-30
Protein binding % (human)⁎	90	77.4	80
Molecular weight (g/mol)	410.48	426.48	371.26

⁎ Data on file.

⁎⁎ Data from Richtand et al. [2]. Values depicted for D_2_ and D_2_ long receptor in vitro experimental *KI*.

**Table 2:** Model parameters used for the simulations in exploratory model analysis. Pharmacokinetic and pharmacodynamic parameters obtained from Kozielska et al. [3] and Stevens et al. [4], respectively.

Parameter	Estimate
*CL* (l·h^-1^)	1.62
*V1* (l)	1.29
*Q* (l·h^-1^)	0.0882
*V2* (l)	0.169
*F*	1
*Ka* (h^-1^)	2.84
*C*_*prl,0*_ (ng·ml^-1^)	6.2
*R*_*form*_ (ng·ml^-1^·h^-1^)	35.3⁎
*K*_*base*_ (h^-1^)	0.57
*K*_*out*_ (h^-1^)	5.7
*E*_*max*_	25
*EC*_*50*_ (ng·ml^-1^)	0.08
*γ*	1
*E*_*max_pf*_	3.5
*EC*_*50_pf*_ (ng·ml^-1^)	12.4

*CL* = clearance from the central compartment, *V1* = volume of the central compartment, *Q* = intercompartmental clearance, *V2* = volume of the peripheral compartment, *F* = bioavailability, *Ka* = absorption constant, *C*_*prl,0*_ = plasma concentration of prolactin in the absence of antipsychotic drug, *R*_*form*_ = zero-order rate constant for prolactin synthesis, *K*_*base*_ = first-order rate constant of prolactin release from the pool, *K*_*out*_ = first-order rate constant of elimination of prolactin from plasma, *E*_*max*_ = maximum increase in the prolactin release from the pool, *E*C_*50*_ = drug concentration at half-maximal effect, *γ* = slope parameter, *E*_*max_pf*_ = maximum prolactin feedback, *EC*_*50_pf*_ = plasma prolactin concentration at half-maximal effect.

⁎ *R*_*form*_ is calculated as the product of *C*_*prl,0*_ . *K*_*out*_ (equation (5) of Taneja et al. [1]).

**Table 3:** Parameter comparison (relative standard error in %) of the pool model with different *ECu*_*50*_ for risperidone and paliperidone vs. single *ECu*_*50*_ and the interaction model with different *KI* for risperidone and paliperidone vs single *KI*.

	Estimates	
Different *ECu*_*50*_	Single *ECu*_*50*_
**Pool Model**	52.1 (12)	48.7 (12)
*R*_*form*_ (ng.ml^-1^×h^-1^)
*K*_*base*_ (h^-1^)	0.251 (12)	0.250 (12)
*K*_*out*_ (h^-1^)	6.96 (14)	6.57 (12)
*E*_*max*_	3.5 FIXED	3.5 FIXED
*ECu*_*50*_ paliperidone (nM)	38.2 (52)	35.1 (51)
*ECu*_*50*_ risperidone (nM)	39.6 (142)
*ECu*_*50*_ remoxipride (nM)	95.2 (41)	94.8 (31)
*γ*	1 FIXED	1 FIXED
IIV *R*_*form*_ (%)	40 (18)	40 (18)
Residual error (%)	50 (7)	50 (7)
Minimization	++	++
Covariance	++	++
Objective Function Value	3454.764	3455.775
**Interaction Model**	23.8 (6)	23.8 (6)
*K*_*in,0*_ (ng.ml^-1^×h^-1^)
*K*_*out*_ (h^-1^)	3.35 (10)	3.34 (9)
*K*_*DA*_ (h^-1^)	5.12 (19)	5.51 (21)
*DAs*_*0*_ (dimensionless)	1000 FIXED	1000 FIXED
*KI* paliperidone (nM)	34.1 (11)	35.7 (12)
*KI* risperidone (nM)	47.7 (20)
*KI* remoxipride (nM)	370 (11)	360 (11)
*γ*	1 FIXED	1 FIXED
IIV *K*_*in,0*_ (%)	40 (22)	40 (22)
Residual error (%)	56 (12)	56 (12)
Minimization	++	++
Covariance	++	++
Objective Function Value	3727.802	3726.086

*R*_*form*_ = zero-order rate constant for prolactin synthesis, *K_base_* = first-order rate constant of prolactin release from the pool, *K*_*out*_ = first-order rate constant of elimination of prolactin from plasma, *E*_*max*_ = maximum increase in the prolactin release from the pool, *EC*_*u50*_ = unbound drug concentration at half-maximal effect, *γ* = slope parameter, IIV = inter-individual variability, *K*_*in,0*_ = basal prolactin release rate, *K*_*DA*_ = first-order turnover constant for hypothetical dopamine, *DAs_0_* = hypothetical scaled dopamine concentration at baseline, *KI* = drug potency parameter.

**Table 4:** Pool model: Comparison of parameter estimates with parameterization of drug effects as *ECu*_*50*_ as compared to *RO*_*50*_ (relative standard error in %).

**Parameter**	**Estimates using*****ECu***_***50***_	**Estimates using*****RO***_***50***_
*R*_*form*_ (ng.ml^-1^×h^-1^)	45.7 (10)	49.8 (10)
*K*_*base*_ (h^-1^)	0.25 (10)	0.226 (11)
*K*_*out*_ (h^-1^)	6.06 (12)	6.96 (13)
*E*_*max*_	3.5 FIXED	3.5 FIXED
*ECu*_*50*_ risperidone/paliperidone (nM)	35.1 (51)	⁎
*ECu*_*50*_ remoxipride (nM)	94.8 (31)	⁎
*RO*_*50*_ (%)	NA	28.7 (27)
*γ*	1 FIXED	1 FIXED
IIV *K*_*out*_ (%)	42.1 (18)	42.3 (18)
Residual error - proportional (%)	47.2 (4)	37.4 (8)
Residual error - additive (ng·ml^-1^)	NE	2.68 (29)
Minimization	++	++
Covariance step	++	++
Objective Function Value	3434.44	3430.56

*R*_*form*_ = zero-order rate constant for prolactin synthesis, *K_base_* = first-order rate constant of prolactin release from the pool, *K_out_* = first-order rate constant of elimination of prolactin from plasma, *E*_*max*_ = maximum increase in the prolactin release from the pool, *EC*_*u50*_ = unbound drug concentration at half-maximal effect, *RO*_*50*_ = receptor occupancy at half-maximal effect, *γ* = slope parameter, IIV = inter-individual variability.

NA = not applicable.

NE = not estimated.

⁎ *KI* risperidone/paliperidone = 2.55 nM, *KI* remoxipride = 370.66 nM (fixed to in vitro experimental values).
